# Recent Research Advances in Double-Strand Break and Mismatch Repair Defects in Prostate Cancer and Potential Clinical Applications

**DOI:** 10.3390/cells12101375

**Published:** 2023-05-12

**Authors:** Damian Jaworski, Bartosz Brzoszczyk, Łukasz Szylberg

**Affiliations:** 1 Department of Clinical Pathomorphology, Collegium Medicum in Bydgoszcz, Nicolaus Copernicus University in Torun, 85-067 Bydgoszcz, Poland; 2 Division of Ophthalmology and Optometry, Department of Ophthalmology, Collegium Medicum in Bydgoszcz, Nicolaus Copernicus University in Torun, 85-067 Bydgoszcz, Poland; 3 Department of Urology, University Hospital No. 2 im. Dr. Jan Biziel in Bydgoszcz, 85-067 Bydgoszcz, Poland; 4 Department of Obstetrics, Gynaecology and Oncology, Chair of Pathomorphology and Clinical Placentology, Collegium Medicum in Bydgoszcz, Nicolaus Copernicus University in Torun, 85-067 Bydgoszcz, Poland; 5 Department of Tumor Pathology and Pathomorphology, Oncology Centre-Prof. Franciszek Łukaszczyk Memorial Hospital, 85-796 Bydgoszcz, Poland

**Keywords:** prostate cancer, double-strand break, mismatch repair, PARP inhibitors

## Abstract

Prostate cancer remains a leading cause of cancer-related death in men worldwide. Recent research advances have emphasized the critical roles of mismatch repair (MMR) and double-strand break (DSB) in prostate cancer development and progression. Here, we provide a comprehensive review of the molecular mechanisms underlying DSB and MMR defects in prostate cancer, as well as their clinical implications. Furthermore, we discuss the promising therapeutic potential of immune checkpoint inhibitors and PARP inhibitors in targeting these defects, particularly in the context of personalized medicine and further perspectives. Recent clinical trials have demonstrated the efficacy of these novel treatments, including Food and Drugs Association (FDA) drug approvals, offering hope for improved patient outcomes. Overall, this review emphasizes the importance of understanding the interplay between MMR and DSB defects in prostate cancer to develop innovative and effective therapeutic strategies for patients.

## 1. Introduction

Among all cancers in men, prostate cancer (PC) is the most common non-cutaneous cancer and the second most common cancer worldwide, with approximately 366,000 deaths and 1,600,000 cases annually [1]. PC is characterized by a variable disease course with either aggressive development and metastasis or slow progression without metastasis. These tumors are graded using the Gleason score [2].

The underlying cause of PC is still under investigation, but studies have shown that both differentiated and stem/progenitor cells have the potential to initiate PC of either the luminal or basal phenotype [3], although there is no conclusive result regarding the clinical and biological relevance of the PC phenotype [4,5,6].

Nevertheless, it is widely accepted that chronic inflammation plays a critical role in the development of PC. Prolonged exposure to oxidative stress and reactive oxygen species can cause DNA damage, leading to the selection of mutated cells and the progression to prostate intraepithelial neoplasia and malignancy [7].

Although numerous studies have identified multiple genetic alternations associated with PC, the genetic and epigenetic features of PC in relation to DNA repair are currently poorly understood. Impaired DNA repair pathways are considered to be the cause of several types of cancers, including XP gene loss in skin cancer, BRCA1/2 defect in ovarian and breast cancers, and mismatch repair (MMR) deficiency in colorectal cancer (Lynch syndrome) [8].

While most PCs occur sporadically, approximately 5–15% are associated with hereditary factors. Studies suggest that mutations in DNA damage repair (DDR) genes, including DNA MMR and double-strand break repair (DSBR) genes, may be used as biomarkers for hereditary PC (HPC), which is characterized by a more aggressive disease course [9,10,11]. DDR is involved in tumorigenesis, but is also an important factor in treatment. The identification of impaired DNA repair pathways in specific cancers is crucial to the exploration of new treatment protocols that exploit DDR defects. This approach has been applied to numerous cancers, including BRCA1/2-deficient ovarian and breast cancers, where the inhibition of poly(ADP-ribose) polymerase 1 (PARP) has been proven to be synthetically lethal in other tumors with PTEN deficiency, and, more recently, in patients with homologous recombination (HR) repair gene-mutated metastatic castration-resistant PC (mCRPC) [8,12,13].

Moreover, DDR is also known to be useful in predicting drug resistance in cancer. In the treatment of O(6)-methylguanine DNA methyltransferase-deficient glioblastomas, increased sensitivity to temozolomide has been observed [14]. In addition, PARP inhibition has been shown to restore temozolomide sensitivity in MMR-deficient tumors [15].

Recent studies have also highlighted the potential role of immune checkpoint inhibitors, such as CTLA-4 and PD-L1 inhibitors, in the context of DDR-deficient tumors [16,17]. These agents unleash the immune system to identify and target cancer cells by exploiting the increased mutational burden and neoantigen load present in DDR-deficient tumors [16,17].

In this review, we have summarized recent studies focused on two of the DDR pathways, MMR and DSBR, in PC and their implications for cancer therapy, particularly in regard to prostate cancer. The aim is to provide a comprehensive overview of the current state of the field and to highlight areas for future research and development.

## 2. Mechanisms of the DNA Damage Repair

DDR is a vital mechanism that cells use to maintain the integrity of their genetic material. DDR is activated in response to various types of DNA damage, such as single-strand breaks (SSBs) repaired by mechanisms such as MMR, base excision repair (BER), nucleotide excision repair (NER), and double-strand breaks (DSBs) that can be repaired by homologous recombination (HR) or non-homologous end joining (NHEJ). By initiating the appropriate repair mechanisms, DDR helps cells to effectively repair DNA damage, thereby preventing replication errors and maintaining genomic stability [18].

## 3. Immune Checkpoint Molecules: PD-L1 and CTLA-4

PD-L1 and CTLA-4 belong to the immune checkpoint molecules that are crucial in the regulation of the immune response against cancer cells. Immune checkpoint inhibitors targeting the CLTA-4 and PD-L1/PD-1 pathways have been proven to enhance anti-tumor immune responses, which indicates a promising strategy for cancer treatment, including for PC [16,17].

Cells with deficiencies in the main DDR pathways, such as MMR and DSBR, accumulate DNA mutations and chromosomal rearrangements, resulting in tumorigenesis and cancer progression [19]. Cancer cells harboring MMR and DSBR defects are characterized by higher mutational burden and increased genomic instability [20]. Furthermore, the aforementioned increased mutational burden results in the generation of tumor-specific neoantigens that could be recognized by the immune system. Consequently, T-cells are activated, which initiates an ani-tumor immunological response. The immune response is stronger in tumors with high mutational burden, which increases the infiltration of such immune cells, such as natural killer cells and T-cells [21,22]. Cancers with MMR and DSBR deficiencies show increased sensitivity to immune checkpoint inhibitors targeting the CTLA-4 and PD-L1/PD-1 pathways as a result of their increased immunogenicity. These cancers, due to the accumulation of mutations, are more susceptible to immune-mediated attack, especially after the inhibition of immune checkpoint molecules [23,24]. Moreover, a combined therapy of PARP inhibitors with immune checkpoint inhibitors could have synergistic effects in cancer treatment [8,25,26,27].

## 4. Implications of the MMR on Tumorigenesis and Its Alterations in Prostate Cancer

MMR is a highly conserved DNA repair that corrects errors in the replication, recombination, and repair processes, preserving genomic stability by correcting mismatched bases, insertion-deletion loop-type mismatches (IDLs), and SSBs [28]. Mutations in MMR genes cause a high frequency of microsatellite instability (MSI), which is characterized by an increased rate of insertion-deletion mutations in repetitive DNA sequences. The severity of this process can be assessed by examining the repetitive sizes of the selected microsatellite markers. Cancers can be defined as MSI-high (MSI-H) (multiple marker instability) or MSI-low (MSI-L) (only one marker instability). MSI is a hallmark of several hereditary and sporadic cancers, including colorectal, endometrial, gastric, ovarian, and pancreatic cancers [29]. Deficient MMR activity leads to the accumulation of DNA damage, genomic instability, and the emergence of cancer-promoting mutations.

MMR defects can occur as a result of germline mutations in MMR genes, somatic mutations in MMR genes, or the epigenetic silencing of MMR gene expression. Germline mutations in MMR genes cause hereditary cancer syndromes, such as Lynch syndrome, which predisposes individuals to the development of colorectal, endometrial, ovarian, prostate, and other cancers [30].

Somatic mutations and the epigenetic silencing of MMR genes occur in sporadic cancers and contribute to the development and progression of cancer. The loss of MMR activity leads to the accumulation of mutations in oncogenes and tumor suppressor genes, as well as the activation of the oncogenic signaling pathways that promote cancer cell survival, proliferation, and invasion [28,31]. This high mutational burden can produce novel tumor-specific antigens, making MMR-deficient tumors more immunogenic and susceptible to immune checkpoint blockade therapies [32]. MMR deficiency has also been associated with an increased sensitivity to certain DNA-damaging agents and PARP inhibitors, as these agents exploit the impaired DNA repair capacity of MMR-deficient cells, leading to synthetic lethality [33,34].

The incidence of MMR defects in PC ranges between 3% and 5% and mainly affects the MSH2 and MSH6 genes [35,36]. In addition to Lynch syndrome, ductal subtypes of PC show a higher number of MMR mutations. This is associated with poorer histopathological differentiation, according to the Gleason score, and a worse prognosis [37,38]. Loss of function alterations in the MMR genes (MLH1, MSH2, MSH6, and PMS2) define a subgroup of patients with a high potential response to immune checkpoint blockade, which together with MSI and a higher expression of tumor neoantigens, facilitates immunological diagnosis. These observations served as the biological basis for testing pembrolizumab (anti-PD1) in high MMR/MSI solid tumors and led to the Food and Drug Administration (FDA) approval of pembrolizumab [39]. However, some patients with the MMR mutations have a high immune resistance, so not all men benefit equally from this treatment [23,40]. Intratumoral DNA sensing deficiency is one mechanism of low T-cell recruitment that can impair the response to immune checkpoint blockade. In MMR tumors lacking a pathway to detect pro-inflammatory cytosolic DNA, tumor growth was accelerated and the immune checkpoint blockade response was lost during treatment with pembrolizumab [41].

Mutations in the MMR genes have been reported in several studies of PC patients. The most commonly altered MMR genes in PC are MLH1, MSH2, MSH6, and PMS2, although other MMR genes, such as MSH3 and PMS1, have also been implicated in some cases [42,43,44,45].

### 4.1. MLH1

MLH1 is an essential component of the MMR system, and its alterations have been associated with MSI and the development of various cancers [46], including increased PC risk [47]. In PC, MLH1 alterations are relatively rare, with a reported loss of MLH1 expression in 0–0.9% of PCs [48,49]. However, in a study by Khan HM et al. in men aged over 75, PCs with a MLH1 mutation had a cumulative incidence of 13.8% [50]. MLH1 alterations in PC have been associated with higher Gleason scores, aggressive tumor behavior, and a poor prognosis [51]. One study suggests that MLH1 deficiency may contribute to resistance to radiotherapy in PC cells, indicating that a patient’s MLH1 status may influence their treatment response [52]. In addition, a study by Rodrigues et al. showed that MLH1-deficient PC cells were more sensitive to the PARP inhibitor olaparib, suggesting a potential therapeutic strategy for patients with MLH1 alterations [53]. Moreover, studies show that a lower expression of MLH1 protein in PC correlates with a higher prevalence of lymph nodes metastases. In addition, these studies suggest a positive correlation between the Gleason pattern and MLH1 protein expression [54,55].

### 4.2. MSH2

MSH2 alterations have also been identified in PC patients. The reported prevalence of MSH2 alterations is estimated to be 1–2% in mCRPC [32,42,56], and a loss of MSH2 expression is estimated to occur in 2.7–12.2% of PCs [48,49]. These alterations include point mutations, deletions, and rearrangements, resulting in a loss of MSH2 protein expression and impaired MMR function [57]. In the study by Hiba et al., patients at the age of 75 with MSH2 mutations had a cumulative PC incidence of 23.8% [50]. Interestingly, Jaworski et al., in their study, indicate a negative correlation between MSH2 nuclear expression in PC and the Gleason pattern, as well as a positive correlation between nuclear and cytoplasmic expression with the Gleason score. [54] MSH2 mutations are associated with a higher risk of developing PC and are implicated in disease aggressiveness and progression [50,58].

### 4.3. MSH6

MSH6 mutations in PC have been reported in several studies. MSH6 alterations have been reported in approximately 1% of PC cases [42,59,60,61]. A loss of the immunohistochemical expression of MSH6 was found in 2.7–16.8% of PCs [48,49]. However, Alberto-Gonzalez et al., in their study, revealed MSH6 overexpression in 42.1% of the cases [62]. In a study by Pritchard et al., MSH6 mutations were found in 0.14% of men with metastatic PC [42]. MSH6 alterations were associated with higher Gleason scores, an advanced stage, and a poor prognosis [62].

### 4.4. PMS2

PMS2 alterations are less common in PC compared to other MMR genes. In a study by Pritchard et al., PMS2 mutations were found in 0.29% of men with metastatic PC [42]. Another study found PMS2 mutations in 0.4% of metastatic PC patients [59]. However, Sharma M. et al. and Javeed S. et al., in their studies, revealed a loss of PMS2 expression in 12.3% and 12.2% of PCs, respectively [48,49]. Although MMR gene alterations are associated with a worse prognosis in PC patients, there are no exact data correlating the PC grade with PMS2 expression [63].

### 4.5. Clinical Implications of MMR Alterations in Prostate Cancer

MMR alterations in PC have significant clinical implications. Patients with germline MMR mutations are at a higher risk of developing PC and other cancers, such as colorectal and endometrial cancers [64,65,66]. MMR alterations are also associated with aggressive tumor behavior, a higher Gleason scores, an advanced stage, and a poor prognosis [67,68]. MMR alterations have been identified as potential biomarkers for treatment response. In a study by Abida et al., patients with mCRPC harboring MMR alterations were more likely to respond to immune checkpoint inhibitors, such as pembrolizumab, compared to those without MMR alterations [36]. MMR deficiency has also been associated with an increased sensitivity to PARP inhibitors, such as olaparib [69].

#### 4.5.1. Impact of MMR Alterations on Treatment Strategies in Prostate Cancer

The presence of MMR alterations in PC has led to the development of targeted therapies and personalized treatment strategies. Two main classes of targeted therapies have shown promise in the treatment of PC patients with MMR alterations: immune checkpoint inhibitors and PARP inhibitors.

#### 4.5.2. Immune Checkpoint Inhibitors

MMR-deficient tumors are characterized by MSI-H and an increased neoantigen load, which makes them more susceptible to immune checkpoint inhibitors [23]. Several clinical trials have reported the efficacy of pembrolizumab and nivolumab, the PD-1 inhibitors, in MMR-deficient PC patients. The KEYNOTE-028 trial demonstrated an objective response rate of 27% in patients with PD-L1-positive mCRPC [70]. The KEYNOTE-199 trial reported a 50% overall response rate in patients with MSI-H- or MMR-deficient mCRPC [71]. Based on these findings, pembrolizumab has been approved by the FDA for the treatment of MSI-H- or MMR-deficient mCRPC patients who have progressed in prior treatment. A summary of the drugs that have been studied thus far in the treatment of prostate cancer with the immune checkpoint inhibitors group can be found in Table 1.

#### 4.5.3. PARP Inhibitors

PARP inhibitors, such as olaparib and rucaparib, both of which are approved by the FDA in mCRPC, target the PARP enzyme involved in DNA repair. PARP inhibitors have shown efficacy in MMR-deficient cancers by exploiting the synthetic lethality, where two independent DNA repair pathways are disrupted, leading to cell death [80]. Although initially developed for BRCA-mutated and homologous recombination repair-deficient tumors, PARP inhibitors have also shown potential in MMR-deficient cancers [81]. Furthermore, it has been proposed that the clinical application of PARP inhibitors in prostate cancer could be broadened by combining them with androgen receptor inhibitors, which have been found to suppress the expression of numerous HR genes [27].

The TOPARP-A trial (NCT01682772) [61] and the TRITON2 trial (NCT02952534) [82] evaluated olaparib and rucaparib, respectively, in patients with mCRPC harboring DNA repair gene alterations, including MMR gene alterations. Both trials demonstrated antitumor activity in patients with MMR-deficient PCs, suggesting a potential role for PARP inhibitors in this patient population. A summary of the drugs that have been studied to date in the treatment of prostate cancer with the PARP inhibitors can be found in Table 2.

#### 4.5.4. Novel Treatment Strategies Aiming MMR Genes

As research progresses, new drugs and strategies targeting MMR genes in PC may emerge. Combining immune checkpoint inhibitors with other immunotherapies, radiation or chemotherapy could potentially enhance their efficacy in MMR-deficient PCs [88]. Additionally, novel small molecules targeting MMR proteins, such as MSH2-MSH6 inhibitors [89], could be developed and tested in PC.

Moreover, biomarker-driven patient selection will be critical in identifying the most appropriate treatment options for individual patients. Comprehensive genomic profiling can help identify MMR-deficient PCs and guide personalized therapy [90].

## 5. Implications of the DSBR on Tumorigenesis and Its Alterations in PC

DSBs are a severe form of DNA damage that can arise from endogenous factors during DNA replication or can be induced by exogenous agents, such as ionizing radiation and chemotherapeutic agents. To maintain genomic integrity, cells have evolved two main pathways for repairing DSBs: HR and NHEJ. These pathways involve a complex interplay of proteins, including DNA damage sensors, signal transducers, mediators, and effectors. [18,29] Moreover, there are three additional mechanisms involved in DSBs repair: alternative NHEJ (alt-NHEJ), break-induced replication (BIR), and single-strand annealing (SSA) [91,92].

BRCA1 plays a critical role in regulating the balance between HR and NHEJ, with a loss of BRCA1 resulting in a shift towards NHEJ and increased sensitivity to DNA damaging agents. 53BP1 and its downstream effector RIF1 are key factors in promoting NHEJ and suppressing HR, with a loss of 53BP1 leading to increased HR and reduced NHEJ. MDC1 acts as a scaffold for the recruitment of DNA damage response proteins, including NBS1, 53BP1, RNF8, the MRN complex, and RNF168, which ubiquitylate histone H2AX and promote the accumulation of 53BP1 and BRCA1 at DSBs. The process of the phosphorylation of histone H2AX is catalyzed by the PI3-like kinase ataxia-telangiectasia mutated (ATM) [19,92,93,94].

The 53BP1/MDC1 axis is a key regulator of the DSB repair pathway choice, with 53BP1 promoting NHEJ and inhibiting HR, while MDC1 promotes HR. This is achieved, in part, by the differential regulation of RPA and RAD51 by the two proteins. 53BP1 inhibits the loading of RAD51 onto the DNA ends, thereby preventing HR, while MDC1 promotes the retention of RPA and the loading of RAD51, thus promoting HR [19,95]. In addition, BRCA1 counteracts 53BP1′s inhibition of HR, while BRCA2 plays a role in the loading of RAD51 onto resected DNA ends [96,97]. Mutations in these genes have been associated with an increased risk of developing cancer, particularly breast and ovarian cancers [98].

Defective DSBR main pathways, such as HR, often coexist with MMR defects, leading to the accumulation of DNA damage and genomic instability [99,100]. As a result, the affected cancer cells become more dependent on alternative DNA repair pathways, such as the ssDNA repair mechanisms [101]. Exploiting the vulnerabilities in these alternative DNA repair pathways can lead to synthetic lethality, selectively eliminating cancer cells while sparing normal cells. This method is used by PARP inhibitors in cancers with MMR and HR defects [61]. PARP inhibitors block the repair of ssDNA breaks, leading to the accumulation of DSBs that the affected cells are unable to repair, resulting in the death of the cancer cell [80].

Alterations in the DSB repair pathway have been implicated in PC progression and treatment resistance. Two primary pathways are responsible for repairing DSBs in eukaryotic cells: HR and NHEJ. Both pathways are crucial for maintaining genomic stability, and defects in either pathway can lead to genomic instability and cancer development [102]. Multiple proteins play a critical role in the DSB repair mechanisms, and alterations in these proteins have been observed in PC. In this section, we will discuss the key proteins associated with both the HR and NHEJ pathways.

### 5.1. Homologous Recombination

The key proteins involved in the HR pathway include BRCA1, BRCA2, RAD51, and the MRN complex (MRE11, RAD50, and NBS1) [94,95,100].

#### 5.1.1. BRCA1 and BRCA2

BRCA1 and BRCA2 are crucial proteins in the HR pathway, and germline and somatic mutations in these genes have been observed in PC [103]. Men with BRCA1 mutations account for 0.9% of PCs and have a 3-fold increased risk, and those with BRCA2 mutations account for 1.2–5.3% of PCs, overall, and have an 8-fold increased risk of developing PC [42,104]. In metastatic PC, the prevalence of BRCA2 mutation is estimated at 13.0% [105]. BRCA1/2 mutations in PC are associated with more aggressive disease, a higher Gleason score, increased metastasis [106], a poor prognosis, and resistance to conventional therapies [42]. As a result of the deficiency in DNA repair, agents such as platinum-based chemotherapy [107] and PARP inhibitors have shown promise in the treatment of BRCA1/2-mutated PCs [33,61,105]. Moreover, two of the PARP inhibitors, rucaparib and olaparib, have received FDA approval for BRCA-mutated mCRPC [108,109].

#### 5.1.2. MDC1

The primary function of MDC1 is involved in the HR pathway of DSBR; it acts as a scaffold protein and recruits other factors at the site of DNA damage in the HR pathway [110]. Studies have revealed the overexpression of MDC1 in various malignancies, although the study by Jaworski et al. shows decreased MDC1 expression in PC with higher GS [54,111,112]. Moreover, MDC1 alteration is associated with the increased radiosensitivity of PC [113], and MDC1 knockdown promotes PC cells’ migration and growth [112].

#### 5.1.3. The RAD Family of Genes: RAD51 and RAD54

RAD51, responsible for catalyzing the strand invasion step and playing a crucial role in homologous recombination repair, has been shown to be affected by the deletion of MMS22L, which is commonly observed (up to 14%) in prostate cancer [114]. In a study by Mitra A. et al., RAD51 cytoplasmic staining was observed in 32.5% of PC cases compared with 0.74% of benign prostate tissues and has been associated with aggressive disease [42,115]. Moreover, RAD51 overexpression in PC is associated with the enhanced sensitivity of PC to radiotherapy [116]. Hine et al. indicated that the inhibition of RAD51 sensitizes PC cells to radiotherapy and chemotherapy [117]. RAD54, another key player in HR, has also been implicated in PC. Genetic alterations in RAD54 have been associated with an increased risk of PC [118].

#### 5.1.4. MRN Complex

The MRN complex, consisting of MRE11, RAD50, and NBS1, plays a crucial role in sensing and repairing DSBs during the HR pathway [119]. Alterations in MRN complex proteins have been reported in PC, with potential implications for disease progression and treatment resistance [119]. MRE11 overexpression correlates with a poor outcome and progression of PC [120]. Alterations in MRN complex proteins, such as MRE11 and RAD50, can affect the sensitivity of PC cells to radiotherapy and chemotherapy with PARP inhibitors [121].

### 5.2. Non-Homologous End Joining

The key proteins involved in the NHEJ pathway include TP53BP1, Ku70, Ku80, DNA-PKcs, and LIG4 [122].

#### 5.2.1. TP53 and TP53BP1

TP53BP1 is a protein that interacts with TP53 and plays an important role in the DSBR pathway, and its primary role is in the NHEJ pathway [123]. This interaction leads to the activation of TP53-dependent cell cycle checkpoints and apoptosis, ensuring the proper cellular response to DNA damage [124]. Alterations in this protein disrupt the function of the NHEJ pathway and promote the utilization of error-prone alt-NHEJ, which can lead to genomic instability and tumorigenesis [125,126]. Studies have shown that mutations in TP53BP1 are present in PC and its expression decreases with cancer progression [127,128,129,130]. In addition, the study by Jaworski et al. indicates no correlation between TP53BP1 expression and GS or GP, while Gzil et al. observed decreased TP53BP1 expression in lymph node metastases compared to primary PC [54,55]. Studies indicate that alterations in TP53BP1 were correlated with the insensitivity of PC to radiotherapy [54,131,132]. Moreover, Chipidza FE et al., in their study, described the mutation of the TP53 gene as an independent, unfavorable prognostic factor in PC [133]. The frequency of TP53 mutations in metastatic PC is estimated to be 31.3% [105].

#### 5.2.2. Ku70 and Ku80

Ku70 and Ku80 are essential proteins in the NHEJ pathway, forming a heterodimer that binds to DSB ends. The impact of alterations in Ku70 and Ku80 expression on PC development is significant, not only because of their direct involvement with DSBR, but also because of their interaction with the androgen receptor as a coactivator [134]. A decrease in the expression of Ku70 has been observed in PC cells following neoadjuvant castration therapy, which in turn impairs DNA repair, and it is suggested as an explanation for the increased sensitivity to radiotherapy in PC following castration [135]. Hasewaga T. et al. suggested that radiotherapy combined with androgen deprivation therapy is effective in patients with GS ≤ 7 or low Ku70 expression [136].

#### 5.2.3. DNA-Dependent Protein Kinase Catalytic Subunit (DNA-PKcs)

The DNA-PKcs is a key component of the NHEJ pathway and plays a critical role in the DNA damage response. The dysregulation of the DNA-PKcs has been implicated in PC progression, metastasis, and resistance to therapy, and its upregulation correlates with poor patient outcomes [60,137,138]. In a study by Pu J. et al., it was suggested that the downregulation of the Androgen receptor/PARP/DNA-PKcs axis could be used as a potential therapeutic strategy to increase the radiosensitivity of castrate-resistant PCs [139].

#### 5.2.4. LIG4

LIG4 is a critical protein in the NHEJ pathway and is responsible for the ligation step during DSB repair [140]. LIG4 has been implicated in PC progression and therapeutic response, including urogenital radiotoxicity [141], although the precise role and clinical significance of LIG4 alterations in PC remain to be fully elucidated [113,141]. High LIG4 expression correlates with advanced GS and nodal involvement [142].

#### 5.2.5. ATM

The ataxia-telangiectasia mutated (ATM) protein is another key player in the DSB repair process through the HR and NHEJ pathways [19,93]. ATM mutations are associated with aggressive PC and occur in 13.7% of metastatic PC [105,143]. Preclinical studies have shown that ATM inhibitors can sensitize PC cells to radiotherapy [144]. The studies conducted thus far have revealed better progression-free survival in mCRPC patients treated with lutetium-177-prostate-specific membrane antigen-617 compared with cabazitaxel, but without an overall improvement in survival [33]. Moreover, alterations in the ATM gene are associated with improved overall survival in PC patients treated with olaparib [33]. However, in mCRPC patients treated with olaparib, there was no objective radiological response, in contrast to BRCA1/2 patients [145]. Interestingly, ATM alterations were correlated with better outcomes to cisplatin-based chemotherapy in patients with mCRPC, compared to mCRPC with CDK12 defects [146].

#### 5.2.6. XRCC1, XRCC2 and XRCC3

XRCC2 and XRCC3 are essential for the RAD51-mediated HR repair of DSBs [147]. Studies have reported associations between XRCC2 and XRCC3 polymorphisms and PC risk, although the evidence is not entirely consistent [141,148,149]. Moreover, polymorphism in XRCC3 is associated with an increased risk of acute genitourinary toxicity during radiotherapy in PC patients [149]. Further research is needed to understand the precise role of these proteins in PC progression and the response to therapy.

## 6. Current Role of DDR Mutation in Prostate Cancer Treatment

Due to the prevalence of germline mutations in the DDR genes, the guidelines of the European Association of Urology (EAU) and the National Comprehensive Cancer Network (NCCN) recommend germline testing for all men with metastatic disease and castration-resistant PC [150,151].

On the other hand, the diagnostic process for DDR mutations should begin at the time of PC diagnosis, especially in patients who meet the criteria for active surveillance (AS) but have a history of familial PC: men with high-risk PC and a family member diagnosed with PC at age < 60 years or a family member who died from PC cancer or familial syndromes, such as hereditary breast and ovarian cancer and Lynch syndrome [152]. The results of the study of 1211 men under AS showed that carriers of the BRCA2 and five ATM mutations were significantly more likely to be reclassified and to progress to clinical disease, requiring exclusion from observation [153]. Interestingly, germline DNA repair gene mutations are not only found in high-risk cancers. The short-term outcomes of AS for low-risk PC showed that at a median follow up of 28 months (IQR 8.5–42), 80% of patients on AS with low-risk PC were free from upgrading or radical treatment [154]. Therefore, patients with DDR mutations included in AS should be carefully monitored until more reliable data are available [151].

The incidence of HR repair mutations in men with PC is significantly higher in the presence of metastases (11% vs. 33% M0/M1) [155]. Furthermore, the Profound study showed that in patients with mCRPC, the number of DDR mutations was lower in the primary tumor (27%) than in the metastatic tissue (32%) [156]. Additionally, the outcomes of this clinical trial revealed a notably longer PFS and a higher ORR for men treated with olaparib compared to the control group, with 7.4 months versus 3.6 months and 33% versus 1%, respectively [157]. In PROREPAIR-B, 68 mCRPC patients with germline BRCA2 mutations had half the CSS compared to non-carriers (17.4 vs. 33.2 months, *p* = 0.027). Importantly, ATM or BRCA1 mutations showed no difference in the CSS in this group of patients [158]. The ability of a cancer cell to repair double-stranded DNA breaks with a BRCA2 mutation is impaired; however, further repair of damage is possible through the activity of PARP. In May 2020, the FDA approved the oral PARP inhibitors rucaparib (Rubraca) and olaparib (Lynparza) for the treatment of mCRPC; talazoparib, niraparib, and veliparib are under investigation. A recent study indicates that PARP inhibitors may be effective not only in BRCA1/2-defficient tumors, but also in tumors with other DDR-deficiencies, such as MMS22L deletion [114]. The efficacy of PARP inhibitors in PC is highest when the number of mutations in the HR repair genes and DSBR is high [159]. In TOPARP-A and TOPARP-B, patients with BRCA1, ATM, PALB2, and FANCA mutations treated with 400 mg of olaparib twice daily achieved clinical benefit (including radiological response, decrease in PSA, and/or reduction in circulating tumor cell count) [61,160]. Moreover, patients with mCRPC and alterations in the DDR genes are more sensitive to platinum chemotherapy, and this is also the case after progression on PARP inhibitors [82]. Importantly, men previously treated with both docetaxel and at least one androgen receptor pathway inhibitor (ARPI) whose tumors had homozygous deletions or deleterious mutations in the DNA repair genes had an 88% response rate to olaparib [61]. A phase III, randomized, double-blind study (PROpel) of abiraterone (1000 mg once daily) plus prednisone 5 mg/twice daily (AAP) and olaparib (300 mg twice daily) in patients with mCRPC showed that the imaging-based progression-free survival (ibPFS) may have been dependent on the number of mutations in the homologous recombination repair gene [161]. Patients who qualified for mCRPC treatment with olaparib must have a mutation in one of the 14 genes, including: BRCA1, BRCA2, ATM, CHEK2, PALB2, and CDK12. Despite the positive results of the treatment of patients with mCRPRC and the ATM mutation in the TRITON2, TRITON3, and GALAHAD preclinical trials, rucaparib cannot be recommended for patients with mutations other than BRCA [82,109,162].

The discovery of an aggressive clinical course, resistance to hormonal treatment, and the occurrence of histological forms with a worse prognosis in patients with mCRPC and the CDK12 mutation prompted researchers to search for a link between MMR deficiency and immune characteristics [163]. Moreover, CDK12 mutations have been observed to occur much less frequently in BRCA2 mutations than in homologous recombination deficiency mutations. Therefore, a different mechanism of association of MMR mutations and high MSI with increased T-cell association with immune checkpoints has been noted [164]. It should be emphasized that the efficacy of using the anti-PD-1/PD-L1 antibody in patients with mCRPC following prior hormonal therapy depends on the number of biallelic CDK12 mutations [163] as both Phase III IMbassador 250 (atezolizumab + enzalutamide) and Phase II STARVE-PC (nivolumab + ipilimumab), which did not test CDK12 expression in patients with mCRPC, failed to meet the primary endpoint of improved overall survival in the unselected patients [71,165]. Interestingly, in the KEYNOTE-199 study, the median OS and disease control rate (DCR) after pembrolizumab were highest in the group of patients with mCRPC with dominant bone metastases, regardless of PD-L1 expression, compared to the selected group of patients with a high expression of these proteins [166]. The lack of conclusive data on the efficacy of the use of pembrolizumab in men with PC has led clinicians to conclude that new treatment strategies are needed to improve the efficacy of CDK12 mutation checkpoint blockade in patients with MMR [167].

## 7. Conclusions

Undoubtedly, the role of the clinical geneticist is becoming increasingly important at the current stage of mCRPC management, and due to the prognostic value of the homologous recombination repair number, the indications for somatic and germline mutation testing in high-risk cancer will expand [168]. DDR mutations can be identified through the analysis of peripheral whole blood testing or tumor tissue. The current objective advantage of tissue testing is the simultaneous analysis of both genomic and somatic mutations. On the other hand, the multifocal and heterogeneous nature of PC in the context of tissue testing may result in the analyzed core biopsy not representing a clone of metastatic disease [169]. Therefore, taking into account the invasive nature of the material collection (visceral, bone metastases) and the 20% false negative rate due to the quality of the material collected, the improvement of blood assessment methods seems promising [161]. The use of liquid biopsy achieves 93% concordance between BRCA 1/2 mutations detected in tissue biopsy and those identified by ctDNA, 100% concordance for germline variants, and the detection of alterations in Tp53, RA, BRCA2/1, PI3K/AKT/mTOR, WNT/β-catenin pathway genes, RAS/RAF/MEK, and MSI-H is also possible [170]. In addition to their predictive value, ctDNA, PacBioScience, and Oxford Nanopore may have a predictive value for patients in active surveillance and salvage therapy; however, the cost and wide availability of genomic profiling tools continue to limit the development of this technology [171,172].

It seems that PC is a heterogeneous group of diseases, heterogeneous in terms of MMR and DSBR deficiency and PTEN protein mutations, which determine different clinical courses and resistance to treatment. Thanks to the improvement of molecular classification and the detailed analysis of MMR, including personalized therapy and targeted treatment at PD-1/PD-L1, PARP inhibitors and future novel treatment strategies will prove to be more effective [173].

## Figures and Tables

**Table 1:** Summary of all completed and selected ongoing clinical trials investigating immune checkpoint inhibitors in the treatment of prostate cancer. Summarized outcomes for all phase 3 clinical trials and selected phase 2 and 1 clinical trials.

Drug Name	Clinical Trial Number	Efficacy/Results		Annotation
Pembrolizumab	NCT02054806 [70] Phase 1	ORR: 17.4%		Investigated in locally advanced and/or metastatic PC
		PFS: 3.5 months		
		OS: 7.9 months		
	NCT03093428 [72] (active) Phase 2	Pembrolizumab + Radium 223	Radium 223	Pembrolizumab + Radium 223 vs. Radium 223 alone in mCRPC
		OS: 16.9 m	OS: 16.0 m	
		PFS: 6.1 m	PFS: 5.7 m	
	NCT02787005 [73] Phase 2	Full description of the results available in the citation		mCRPC patients divided into 5 cohorts. Accelerated FDA-approval in May 2017 for unresectable/metastatic, MSI-H or MMR-deficient solid tumors
	NCT03658447 NCT03582475 NCT04148937 NCT03849469 Phase 1			
Nivolumab	NCT02601014 [74] Phase 2	Nivolumab + Ipilimumab	Enzalutamide + Nivolumab + Ipilimumab	Investigated in mCRPC
		ORR: 25%	ORR: 0%	
		OS: 8.2%	OS: 14.2%	
		PFS: 3.7 m	PFS: 2.9 m	
	NCT03554317 NCT00441337 NCT03532217 NCT03835533 Phase 1			
Atezolizumab	NCT03016312 [75] Phase 3	Atezolizumab + enzalutamide	Enzalutamide	Investigated in combination with enzalutamide in mCRPC
		OS: 15.2 m	OS: 16.6 m	
		rPFS: 4.2 m	rPFS: 4.1 m	
		OR: 13.7%	OR: 7.4%	
	NCT04404140 NCT03024216 NCT02814669 NCT02655822 Phase 1			
Durvalumab	NCT04089553 [76] (active) Phase 2	AZD4635 + Durvalumab	AZD4635 + Oleclumab	Investigated in combination with AZD4635 in mCRPC
		% of patients with rPRFS at 6 months: 8.8%	% of patients with rPRFS at 6 months: 11.1%	
	NCT03204812 [77] Phase 2	rPFS: 3.7 m OS: 28.1 m		Durvalumab + Tremelimumab in naive patients with mCRPC
	NCT04495179 Phase 2, NCT02643303 Phase 1			
Ipilimumab	NCT00861614 [78] Phase 3	Ipilimumab + RTH	Placebo + RTH	Investigated as monotherapy or in combination with RTH in mCRPC
		OS: 11.04 m	OS: 10.02 m	
		OSR at year 5: 7.9%	OSR at year 5: 2.7%	
		PFS: 4.01 m	PFS: 3.06 m	
	NCT01057810 [79] Phase 3	Ipilimumab	Placebo	
		OS: 28.65 m	OS: 29.73 m	
		PFS: 5.59 m	PFS: 3.81 m	
	NCT01194271 NCT02279862 NCT00323882 NCT00170157 NCT00050596 NCT02601014 NCT01498978 NCT01804465 Phase 2 NCT03532217 NCT00064129 NCT02113657 NCT00323882 NCT01832870 Phase 1			

Abbreviations: ORR: Overall response rate; OS: overall survival; OSR: overall survival rate; rPFS: Radiographic Progression-Free Survival (in months); PFS: Progression-Free Survival (in months); m: months; PC: prostate cancer; mCRPC: Metastatic Castration-Resistant Prostate Cancer.

**Table 2:** List of all completed and selected ongoing clinical trials investigating PARP inhibitors in the treatment. Summarized outcomes for phase 2 and 3 clinical trials if available (NCT02987543, NCT02952534, NCT02854436, NCT03148795, and NCT01576172).

Drug Name	Clinical Trial Number/Phase	Efficacy/Results					Annotation
Olaparib	NCT02987543 [83] Phase 3	Cohort A with olaparib	Cohort A with Investigators Choice of NHA	Cohort B with olaparib	Cohort B with Investigators Choice of NHA		Approved by FDA for mCRPC with HRR gene alterations, including BRCA1/2, ATM (PROfound clinical trial). Cohort A: mCRPC with either BRCA1/2/ATM mutation Cohort B: 12 other genes involved in the HRR. Investigators Choice of NHA: enzalutamide or abiraterone acetate
		ORR: 33.3%	ORR: 2.3%	n/a	n/a		
		OS: 56.2%	OS: 68.7%	n/a	n/a		
		RPFS: 7.39 m	RPFS: 3.55 m	n/a	n/a		
		RPFS in cohort A + B with olaparib: 5.82 m		RPFS in cohort A + B with Investigators Choice of NHA: 3.52 m			
	NCT03205176, NCT02324998 Phase 1 NCT03434158 Phase 2						
Rucaparib	NCT02952534 [84] Phase 2	BRCA	ATM	CDK12	CHEK2	Others	Approved by FDA for mCRPC with BRCA1/2 mutations that have been previously treated with androgen receptor-directed therapy and a taxane-based chemotherapy (TRITON2 clinical trial) mCRPC patients divided into 5 groups with: either BRCA, ATM, CDK12, CHEK or other HRR gene mutation.
		ORR: 45.7%	ORR: 0%	ORR: 0%	ORR: 0%	ORR: 41.2%	
		RPFS: 10.7 m	RPFS: 5.3 m	RPFS:3.7 m	RPFS: 9.4 m	RPFS: 11.6 m	
		OS: 17.2 m	OS: 14.6 m	OS: 13.9 m	OS: 11.1 m	OS: 11.6 m	
	NCT03840200 Phase 1						
Niraparib	NCT02854436 (active) Phase 2	ORR: 50%; mPFS: 11 months [85]					Investigated in combination with abiraterone and prednisone in mCRPC with HRR gene alterations, including BRCA1/2, ATM
	NCT02924766 Phase 1b NCT03076203, NCT00749502 Phase 1						
Talazoparib	NCT03148795 Phase 2 (active) [86]	ORR: 29.8% PFS: 5.6 m					Investigated in mCRPC who previously received taxane-based chemotherapy and progressed on at least 1 novel hormonal agent
	NCT03330405 Phase 2	Investigated in solid tumors including PC					
	NCT01286987 Phase 1						
Veliparib	NCT01576172 [87] Phase 2	Abiraterone Acetate + Prednisone		Abiraterone Acetate + Prednisone + Veliparib			Investigated in combination with abiraterone in mCRPC
		ORR: 45%		ORR: 52.2%			
		PFS: 10.1 m		PFS: 11.0 m			
	NCT01085422, NCT00892736 Phase 1						

Abbreviations: NHA: new hormonal agent; ORR: Overall response rate; RPFS: Radiographic Progression-Free Survival (in months); PFS: Progression-Free Survival (in months); M: months; PC: prostate cancer; mCRPC: Metastatic Castration-Resistant Prostate Cancer; HRR: homologous recombination repair; n/a: not applicable.

## Data Availability

Not applicable.
